# “Would a man smell a rose then throw it away?” Jordanian men’s perspectives on women's breast cancer and breast health

**DOI:** 10.1186/1472-6874-13-41

**Published:** 2013-10-25

**Authors:** Hana Taha, Raeda Al-Qutob, Lennarth Nyström, Rolf Wahlström, Vanja Berggren

**Affiliations:** 1 Department of Public Health Sciences, Karolinska Institutet, Stockholm, Sweden; 2 Department of Medical Epidemiology and Biostatistics, Karolinska Institutet, Stockholm, Sweden; 3 Jordan Breast Cancer Program, Amman, Jordan; 4 King Hussein Cancer Foundation, Amman, Jordan; 5 Women and Child Health Division, Department of Family and Community Medicine, University of Jordan, Amman, Jordan; 6 Division of Epidemiology and Global Health, Department of Public Health and Clinical Medicine, Umeå University, Umeå, Sweden; 7 Family Medicine and Clinical Epidemiology, Department of Public Health and Care Sciences, Uppsala University, Uppsala, Sweden; 8 Faculty of Health Sciences, Lund University, Lund, Sweden

## Abstract

**Background:**

Breast cancer is the most common malignancy afflicting women, and the most common cancer overall in Jordan. A woman’s decision to go for screening is influenced by her social support network. This study aims to explore Jordanian men’s individual and contextual perspectives on women’s breast cancer and their own role in the breast health of the females within their families.

**Methods:**

An explorative qualitative design was used to purposively recruit 24 married men aged 27 to 65 years (median 43 years) from four governorates in Jordan. Data in the form of interviews transcriptions was subjected to qualitative content analysis.

**Results:**

Three themes were identified: a) Supporting one’s wife; b) Marital needs and obligations; c) Constrained by a culture of destiny and shame. The first theme was built on men’s feelings of responsibility for the family’s health and well-being, their experiences of encouraging their wives to seek health care and their providing counselling and instrumental support. The second theme emerged from men’s views about other men’s rejection of a wife inflicted by breast cancer, their own perceptions of diminished femininity due to mastectomy and their own concerns about protecting the family from the hereditary risk of breast cancer. The third theme was seen in men’s perception of breast cancer as an inevitable act of God that is far away from one’s own family, in associating breast cancer with improper behaviour and in their readiness to face the culture of Eib (shame).

**Conclusions:**

Jordanian men perceive themselves as having a vital role in supporting, guiding and encouraging their wives to follow breast cancer early detection recommendations. Breast health awareness campaigns could involve husbands to capitalize on family support.

## Background

Breast cancer is the leading cause of cancer deaths in Arab countries [1-3]. Advanced stages of breast cancer are very common and mastectomy is performed in more than 80% of the cases [1]. Thus, it is essential for these countries to implement strategies that create awareness about breast cancer, address modifiable risk factors and enhance early detection [4].

Breast cancer is the most frequently diagnosed cancer in Jordan. In 2009, it accounted for 37% of female cancer cases and 23% of the deaths [5]. The Jordan Breast Cancer Program (JBCP) was established in 2007 as a national initiative led by the King Hussein Cancer Foundation (KHCF) to drive the transformation of Jordanian women’s breast health-seeking behaviour through increasing public awareness about the importance of early detection examinations for breast cancer in saving women’s lives [6].

The JBCP conducts national breast health awareness campaigns and works with all the stakeholders in Jordan to enhance women’s access to quality screening services [6]. Jordan’s national breast health guidelines promote breast health awareness to all Jordanian women including monthly practice of breast self-examination (BSE) starting from the age of 20 years [7]. Clinical breast examination (CBE) is recommended once every 1–3 years in the age group 20–39 years and annually in women aged 40 years or more. Mammography is recommended once every 1–2 years starting from the age of 40 years [7].

To better understand the socio-cultural barriers to breast health-seeking behaviour among Jordanian women, a previous study was conducted by Taha et al. [7] that aimed to explore Jordanian women’s views and perceptions about breast cancer and breast health. The findings showed that women had fears about breast cancer, but they also felt safe and not at risk of the disease. They prioritized the needs of children and family above their own health and mentioned that receiving encouragement from the husband and other family members motivated them to seek early detection of breast cancer. Thus, this study aims to explore Jordanian men’s individual and contextual perspectives on women’s breast cancer and their own role in the breast health of the females within their families.

## Methods

### Study setting

Jordan is an upper-middle-income country with a population of 6 million. In 2010, the annual per capita share from the gross domestic product was 4512 USD [8]. The kingdom has free of charge mandatory educational system for the first 10 years [9,10]. The girls’ primary school enrolment ratio is approximately 96% that of boys [9]. The illiteracy rate among women aged 15 years and above is 10% in comparison with 4% among men. It is a paradox that despite the high literacy rate of Jordanian women, there is a clear difference in the percentage of working women compared to men; in 2010 women constituted 16% of the work force [9,10].

Jordan has three administrative regions representing the north, middle and south with a total of 12 governorates. The interviewees in this study were recruited from the clients of three urban and two rural public comprehensive primary health care (PHC) centres in four governorates: Amman, Balqa, Irbid and Karak. We chose these governorates because they represent Jordan’s three administrative regions and constitute approximately 70% of Jordan’s total population [9]. Amman and Balqa are located in the middle region with a population of 2.4 million and 410 000 respectively [9]. We included in our sample interviewees from both the rich west side of Amman and from the underprivileged east side to capture the possible variations in men’s perceptions and beliefs due to the socio-economical gap between the two parts of the city [11]. Irbid is located in the northern region with a population of 1.1 million and Karak is located in the southern region with a population of 238 000 [9].

There are 67 functional mammography units in Jordan that are inequitably distributed with higher coverage in urban areas; 28 of them are in the public sector, 31 in the private sector, two in the royal medical services, two in King Hussein cancer centre and four in university hospitals [7]. Breast cancer early detection services are provided for free within the Ministry of Health (MoH) civil health insurance system [7]. PHC services in Jordan are well accessible and highly subsidized by MoH. There is a wide coverage nationwide with a density of 2.3 PHC centres per 10 000 capita and accessibility level of approximately 97% [12]. CBE services are available at the comprehensive PHC centres and maternal and child health centres. The centres from where we recruited the interviewees provide CBE screening services, general health care services, family health, maternal and child health care, gynaecology, paediatric, internal medicine, dermatology, ophthalmology, general surgery and dentistry. There is no appointment system for doctors’ consultations at these centres; clients usually walk in and wait for their turn based on first come first served basis.

### Study design

We used individual semi-structured interviews to explore men’s individual and contextual perspectives on women’s breast cancer and their own role in the breast health of the females within their families. Individual interviewing was the most suitable methodology for this study because breast cancer is considered a sensitive subject in Jordan and the research team wanted to ensure the privacy and the confidentiality of the interviewees. The interviews enabled the interviewer (first author) to explore the men’s perceptions and allowed the opinions of the respondents to emerge freely in a pattern that was not predetermined by the interviewer [13].

### Study population

To gather rich and profound data, 24 Jordanian men aged 27 to 65 years (median age 43 years) were recruited purposively [14]. The wide span of the interviewees’ age was because we took into consideration Jordan’s national guidelines of women’s breast health [7]. Our inclusion criteria were: Jordanian married man aged between 20–65 years, and do not have any first degree female relatives who had a previous diagnosis of breast cancer (wife, sister, mother, and daughter). We interviewed twenty four married men, 3 in the age group 20–29 years old, 7 were 30–39 years, 6 were 40–49 years, 4 were 50–59 years and 4 were aged 60–65 years.

The interviewer approached males in the waiting area of the MoH public comprehensive PHC centres who were either patients waiting to see the doctor or were escorting another family member. After introducing herself, the interviewer gave a verbal and a written description of the research and the candidate interviewees who met the inclusion criteria and voluntarily accepted to be interviewed were asked to sign an informed written consent. Four of the men that were approached and met the inclusion criteria declined to be interviewed: one in Irbid, one in Karak and two in west Amman. The socio-demographic characteristics of the interviewees are shown in Table 1.

**Table 1:** The socio-demographic characteristics of the 24 study participants

**Characteristic**	**Category**	**Number**
Age (years)	20-29	3
	30-39	7
	40-49	6
	50-65	8
Governorate	Amman	
	*- East*	5
	*-West*	4
	Balqa'a	6
	Irbid	4
	Karak	5
Education	Primary	6
	High school	8
	College/University	10
Income (JNAAFPL)*	<1	11
	1-2	11
	≥3	2

*JNAAFPL **=** Jordan National Average Absolute Family Poverty Line (food and non-food poverty) in 2008 amounted to 5475 USD annually for the average family size of 5.7 members. [http://www.dos.gov.jo/dos_home_a/main/Analasis_Reports/poverty_rep/poverty_rep_2008.pdf].

### Ethical considerations

This study was conducted according to the ethical principles of the World Medical Association Declaration of Helsinki [15]. It was approved in February 2009 by the Jordan MoH Research Ethics Committee. The participants were informed of the purpose the study, the voluntary nature of their participation and their right to access findings. They had the full autonomy and freedom to change their minds and withdraw at any time, without giving a reason. All the participants were informed about their right not to reveal any personal information. The interviewees were also informed about the possible benefits and harms of their participation in this study before giving a written consent.

From the ethical point of view this study does not carry any serious harm for the interviewees who did not have anyone within their direct family diagnosed with breast cancer. However, there was a possibility that the explorative open-ended questions that invited the participant to talk about breast cancer may have caused anxiety feelings or worries. To sooth any possible fears of that kind, directly after each interview, the interviewer conducted a brief educational session about breast cancer and early detection examinations and distributed educational brochures. The confidentiality of the data was ensured; any sharing of the data within the research team included only numeric codes so that no individual participant could be traced.

### Data collection

The research team prepared an interview guide with open-ended questions to encourage the interviewees to talk freely and spontaneously. The semi-structured interviews were conducted during the period from January to March 2011. Two pilot interviews were conducted in January 2011: the interviewer in the first one was a Jordanian male researcher and in the second one was the first author who is a Jordanian female public health researcher with experience in qualitative research methods. The two interviewees were from west Amman and Balqa respectively. Before these two interviews, both men indicated clearly that they prefer to be interviewed individually and declined to participate in any group discussion. They perceived that it would be inappropriate to share with other men their perceptions about the breast health-seeking behaviours of the females within their families. The second interviewee felt more comfortable and talked to the female interviewer freely without any of the restrictions that the first interviewee displayed while talking to the male researcher. Following these pilot interviews the guide was revised according to the findings and the research team decided that the first author should conduct all the interviews. The interview guide is shown in Table 2.

**Table 2:** Guide of the semi-structured interview

**Domain**	**Questions**
**Perceptions about breast cancer**	• What comes to your mind when you think about breast cancer? *Why?*
	• Do you think that the women within your family (wife, mother, sister, and daughter) are susceptible to breast cancer? *Why?*
	• Did you ever know or hear about a woman who had breast cancer? *In your context, what could happen to a woman who gets diagnosed with breast cancer?*
**Role in women's breast health**	• Can the woman protect herself from breast cancer? *How? Did you previously hear or see anything about breast cancer early detection? What? Where?*
	• What is your role in the health of the women within your family? *Do the women within your family comply with periodic breast examinations? How do you feel about that?*

Each interview was carried out in a quiet room in the health centre and lasted from 20–40 minutes. The interviewer probed to get spontaneous responses from the interviewees and avoided influencing or judging their views [16]. All the interviews were conducted in Arabic and were face to face with each individual interviewee without being escorted by any of their family members. The interviews were audio-taped and an Arabic speaking female research assistant attended to observe and take notes. The tape-recorded data from all the interviews including the pilot ones were transcribed in Arabic and thereafter six of them were translated into English for analysis by the English-speaking co-researchers. Based on the interviews’ accumulating information, the interviewer stopped when information redundancy occurred in each governorate and data saturation was reached at 24 interviews [17,18].

### Data analysis

The transcriptions were read word by word several times by the research team and the data was subjected to qualitative content analysis [19]. The first author condensed the Arabic text into meaning units followed by English coding and categorization. The coding and categorization of the data was validated by all the co-researchers. Thereafter, the research team, in consensus, constructed themes from those categories. The trustworthiness of this study was enhanced through the triangulation of researchers [20].

### Findings

Three themes emerged during the analysis: *First theme:* Supporting one’s wife; *Second theme:* Marital needs and obligations; and *Third theme:* Constrained by a culture of destiny and shame. All the themes and categories are listed in Table 3, the themes are written in bold and the categories in bold-italic. The quotations of the interviewees were labelled using the interviewee number followed by the letters A, B, C, D or E for the governorates; different letter codes were used for Amman’s east and west sides.

**Table 3:** Themes and categories of men’s perceptions about breast cancer and their role in women's screening decisions

**Themes**	**Categories**
Supporting one’s wife	*- Feeling responsible for the family’s health and wellbeing*
	*- Encouraging the wife to seek health care*
	*- Providing counselling and instrumental support*
Marital needs and obligations	*- Other men’s rejection of a wife inflicted by breast cancer*
	*- Diminished femininity due to mastectomy*
	*- Concerns about protecting the family from the hereditary risk of breast cancer*
Constrained by a culture of destiny and shame	*- An inevitable act of God that is far away from one’s own family*
	*- Associating breast cancer with improper behaviour*
	*- Feeling ready to face the culture of Eib*

### Theme 1: supporting one’s wife

The first theme was built on the interviewees’ feelings of responsibility for the family’s health and well-being, their experiences of encouraging the wife to seek health care and their providing counselling and instrumental support.

#### Feeling responsible for the family’s health and well-being

The interviewees talked about breast cancer as a concern not only for women but also for men, since this disease might afflict their wives, daughters, sisters or mothers. Overall, men used religion as a reference point for their compassion towards their families. They viewed marriage as a holy relationship and believed that the husband is accountable for the family’s health and well-being.

“We are in this world to worship God and raise happy and healthy families that avoid diseases … until now, thank God, I have preserved my family, if they complain of anything I would take them to the health centre immediately” (7C)

“If a needle pricks my wife or any of my children I would not wait, I would take them to the doctor immediately” (15B)

The respondents expressed their beliefs that a man and his wife are one entity and talked about the precious value of the wife. They pointed out that the husband is responsible for counselling and guiding the wife to protect herself from breast cancer and for supporting and treating her if she gets inflicted with breast cancer. They mentioned that a married woman should practice breast health examinations because if she neglected her health the negative consequences will affect her husband and children. They spoke about the wife as being an embodiment of the home; meaning if a mother gets afflicted with breast cancer, the tranquillity of family life would be disrupted and her children and husband will suffer as well.

“The wife has her status and respect even more than the man as she is the one who nurtures the young generation while the man is busy outside the home…she is the core of the family, her health is a higher priority than my health” (23E)

#### Encouraging the wife to seek health care

Most of the interviewees viewed breast cancer as a treatable disease that can be cured if detected at early stages. They talked about hearing from the media that early detection is the best way to combat breast cancer.

“As we hear in the media, early detection is the best way to control breast cancer…it leads to a complete cure” (7C)

The respondents spoke about their experience of encouraging their wives to get more information about breast cancer and to seek early detection examinations. They said that a rational woman will do the breast examinations then leave the rest to Allah almighty.

“My wife went to the doctor recently and had a breast examination, she was afraid but I encouraged her, I told her it is better to go and face your doubts and fears, even if God has decided to test you with breast cancer, you will be able to discover the disease early when it is easily treatable” (2A)

“I advised my wife to go to the health centre, where they held lectures for women and taught her how to practice self-breast examination” (8C)

The interviewees had had experience of trying to convince their wives to seek breast examination and they said that the reasons for women reluctance could be shyness, fear, and lack of knowledge or negligence.

“I wish she would ask me to take her to the doctor, but she doesn’t like to go, I encourage her but she refuses as she is afraid that he might discover she has the disease”(23E)

“Some women don’t go for a check-up at the clinic … they are afraid of finding cancer, like my mother…in my opinion, she should be examined for the sole purpose of confirmation… whether she had it or not” (1A)

The interviewees pointed out that BSE is important as it enables the woman to seek advice from a doctor as soon as she discovers any changes in her breasts.

“If she is doing self-examination, she can recognize if she has a lump as soon as it occurs and seek a doctor’s advice, this will save her life” (17B)

All the interviewees preferred that the females within their families get their CBE done by a female doctor; however, the majority accepted a male doctor conducting CBE in the husband’s presence, if a female doctor was not available.

“If there are no female doctors available at all, I would allow a male doctor to do the breast examination for my wife; this would be the last resort” (8C)

#### Providing counselling and instrumental support

The respondents felt that they have an instrumental role in their wives’ health. They explained that their wives consult them prior to seeking breast health examinations and that they give advice and guidance. They also said that they drive or accompany their wives or give them money to go to the health centre. Few men mentioned that their wives are not used to leave home unless being escorted by the husband or older sons.

“She asks my opinion before going to the doctor, so I give her money and tell her to go to the doctor” (15B)

“I bring her to the health centre as we live away from the health centre, how else can she come here? … Her sons also bring her to the centre when she wants” (13B)

Some men said that their wives are doing BSE and as long as everything is normal, the wife does not talk to the husband. However, as soon as she notices any abnormality in her breasts she consults the husband about going to seek a doctor’s opinion.

“My wife doesn’t tell me if she does not suspect anything abnormal, as she practices self-examination…however, when she feels anything strange, she consults me and ask me to take her to the doctor”(20D)

Few men commented on their experiences as sons and their being consulted by the mother about breast health or driving her to the health centre.

“If my mother had mammography I would’ve known.., since it is me who drives her to the hospital” (21D)

### Theme 2: marital needs and obligations

The second theme emerged from the interviewees’ views about other men’s rejection of a wife inflicted by breast cancer, their own perceptions of diminished femininity due to mastectomy and their own concerns about protecting the family from the hereditary risk of breast cancer.

#### Other men’s rejection of a wife inflicted by breast cancer

The interviewees said that fear of breast cancer as a threat that might lead to family destruction and the loss of a loved one might make the man feel helpless and he may not cope with the financial and psychological burdens if the wife was afflicted with breast cancer. They explained that if the man cannot afford the treatment of his wife’s breast cancer, he might have a negative reaction towards her. Since these feelings of helplessness could undermine his role as the head of the family who is responsible for taking care of his wife. Some men mentioned that the man sympathizes with his wife at the beginning of the illness, but with time he changes his position and starts to ignore her and search for another marriage because she is ill and doesn’t satisfy his needs.

“It is a financial burden on the man in addition to other psychological aspects. My aunt had breast cancer she became very ill suddenly…she suffered …and died, her husband wanted to do millions of things but could do nothing. However, the mother of my friend had breast cancer and had a wealthy family that could afford the treatment and taking care of her (1A)

“It is tough on the man ….. how can he cope with this woman who now has a defect in her body, usually, instead of standing by the wife who needs his support to be able psychologically to fight her illness, frequently the man rejects this wife and marries another one who can satisfy his needs” (9C)

“He begins to sympathize with his wife, and then he starts to change his position, ignores her and looks for another marriage. Because she is ill and she cannot satisfy his needs” (8C)

The respondents felt that the good man who is a believer in God’s fate supports his wife if she gets breast cancer and such a test from God might strengthen their marital relation and maximize his compassion towards her. They talked about some husbands within their social context who coped with the burden of the wife’s breast cancer and supported her all through the treatment process until recovery. They also spoke about others who had had a negative reaction and rejected the breast cancer inflicted wife.

“The good man would support his wife and stand by her; he doesn’t let her suffer alone, would a man smell a rose then throw it away? A wife should not be thrown away, she must be protected and have a holy status. I know a man who supported his wife when she had breast cancer because he felt that she belongs to him and considered her the jewel of his life” (7C)

“The man must guide and treat his wife and stand by her until she gets well, not react negatively and not reject her because he can’t handle the burden of her illness” (23E)

#### Diminished femininity due to mastectomy

Most of the interviewees perceived breast cancer as a dangerous disease that can be fatal if detected late. They talked about mastectomy and its consequence on the marital relation.

“If the woman age is above 40 years and had mastectomy, it might be acceptable. However, if she is young it is difficult to accept…if she is unmarried this will influence her marriage chances, and if she is already married, this will influence her marital relation and influence her ability to breast feed her kids” (26E)

However, few young men associated mastectomy with female body deformation. The same young men expressed that a woman afflicted with breast cancer will become less of a woman and deficient.

“It means that there is a defect in her body, if she has had a mastectomy, she will be done with as a female, almost half of her femininity would be gone”(8C)

“If the woman’s breast is removed, this will cause a defect that is difficult for the man to accept as it is not something normal” (21D)

Furthermore, a few men mentioned that the psychological burden of mastectomy on the woman augments her feeling of difference from other women who pity her situation.

“It is very tough on a woman, as she is a female; she needs to feel complete and a whole person, without anything missing. If her breast is removed …she will be different from other women, her husband might reject her and she will be isolated in the society, women will start talking about her breast being removed. She will feel less of a woman in comparison to other women” (9C)

#### Concerns about protecting the family from the hereditary risk of breast cancer

Most of the respondents believed that breast cancer is hereditary and said that a man should better avoid marrying a girl who has a family history of breast cancer. Some explained that, since the man has the choice to select a wife, who will be the mother of his future children, he better seek perfection. On the other hand, a few men commented that breast cancer is not hereditary and a family history of breast cancer should not affect a woman’s chances of marriage.

“If her mother was inflicted with breast cancer then we would not take the daughter for marriage” (13B)

“A man should investigate about hereditary diseases within the family of the girl before marriage and avoid marrying a girl with breast cancer in the family” (5A)

### Theme 3: constrained by a culture of destiny and shame

The third theme was seen in the interviewees’ perception of breast cancer as an inevitable act of God that is far away from one’s own family, and in associating breast cancer with improper behaviour and in their readiness to face the culture of Eib (shame).

#### An inevitable act of God that is far away from one’s own family

Most of the interviewees felt that the women in their families were at risk of breast cancer since illness is an act of God and no one can stop destiny. However, they mentioned that it is a remote risk since they do not have a family history of breast cancer or because their wives were practicing BSE and never had pain or noticed any abnormal changes in the breast. They explained that having no symptoms and no pain means that the woman is safe and there is no need for worry.

“Breast cancer is a disease like other diseases, all women are vulnerable, it is God’s will, it is a test from God and therefore a person should accept it” (18D)

“My wife doesn’t have any symptoms, if she examined herself and found any symptoms, I would take her to the doctor immediately, but no symptoms means that there is nothing to worry about” (14B)

Breastfeeding emerged as a protector or as a predisposing factor for breast cancer. Some men said that a woman who breastfeeds her children protects herself from breast cancer. On the other hand there were a few men who said that breast cancer might happen if the woman stopped breastfeeding suddenly, or if she did not take care of her physical hygiene.

“The woman who has babies and breastfeeds, God protects her from breast cancer at least until she ends that phase” (21D)

“When the woman is breastfeeding and her clothes are not clean, bacteria may pass through the milk and this may lead to breast cancer” (5A)

#### Associating breast cancer with improper behaviour

A few men said that within their context, breast cancer is perceived to be contagious and could be transmitted to the husband. It was pointed out that this perception depends on the husband’s educational level. A few other men commented that it is unfair to women that breast cancer causes repugnance in society and thought that it should be viewed similarly to other diseases. In three interviews men associated breast cancer with promiscuous behaviour. They said that a husband will feel ashamed and reject his wife to avoid catching the illness himself. One man with primary level education perceived that breast cancer is caused by doing things prohibited by Allah (God), such as taking drugs and alcohol.

“Breast cancer comes from promiscuous behaviour; if my sister or aunt gets breast cancer I will disown her as she has brought shame to the family” (28C)

“Some think it is contagious and if a man’s wife is inflicted with breast cancer, this would bring shame to him and his family” (7C)

#### Feeling ready to face the culture of Eib

The interviewees spoke about the society’s pitiful view towards women inflicted with breast cancer. They explained that Jordan is a tribal society and cancer by itself is a taboo word that is avoided because it is seen as a fatal disease. The tribal nature of the society was also brought up within the context that although nowadays women might go to the examination alone, there are still women who cannot go on their own and if the woman tried to tell her husband that she has breast cancer symptoms, he might avoid taking any action to avoid being stigmatized.

“For me it is a disease like any other disease and one should cope with God’s act, however most of the people look with pity towards a woman inflicted with breast cancer” (2A)

“We live in a tribal society. Nobody talks about this subject. If anybody is inflicted with cancer, they would just say, he is ill, because the word cancer is horrific, a reason for late detection might be the husband’s denial, he prefers to keep the problem hidden because he belongs to a tribal society” (8C)

Some men talked about the culture of Eib (shame) and that breast cancer is perceived as a sensitive subject that is not openly discussed in the society because of embarrassment. In Jordan, the culture of Eib can be described as violation of the societal values or norms that could bring disgrace and shame to the individual society member, to his direct family and possibly to his tribe depending on the type and the magnitude of the individual’s inappropriate conduct.

“We have the culture of Eib in our society, you feel shy when there is both fear and shame………but a person should have some courage and decide to go because there is nothing to be afraid or ashamed of” (7C)

Men also mentioned that shyness inhibits the woman from talking about breast cancer and from seeking breast examinations; only two men mentioned that their daughters might talk to them directly if they had any symptoms in the breast. Most of the men said that the daughters talk to their mothers and the mothers tell the father if the girl has any sensitive women’s health issue.

If there is a sensitive women issue like this, my daughters tell their mother, they feel shy to talk to me, but the mother tells me (22D)

The interviewees said that the awareness campaigns about breast cancer helped both women and men to acquire knowledge about the disease and that this contributed to breaking the silence about breast cancer and to challenging the culture of Eib.

“As an eastern society, the majority of us feel sensitive to this matter; some people are embarrassed to talk about this subject. However, I noticed billboards saying “We have had an examination, have you?” … wherever you go, you would hear them talking about the same thing….now everyone has the courage to talk about it, no more embarrassment” (1A)

## Discussion

The interviewees in this study perceived themselves to have a vital role in supporting their wives to follow breast cancer early detection recommendations. They viewed other men’s rejection of a wife inflicted by breast cancer as a failure of the husband to meet the burden of his obligations towards the sick wife and a failure of the sick wife to meet the husband’s marital needs. Men voiced their concerns about breast cancer being a taboo subject in Jordan’s tribal society and appreciated the contribution of breast health awareness campaigns to their talking openly about the disease and to their readiness to face the culture of Eib (shame).

The respondents talked about encouraging their wives to follow breast cancer early detection recommendations. They said that they give advice, guidance or accompany the wife to the health centre or give her money to go on her own. This confirms the findings of our previous study in which women indicated that receiving encouragement from husband and family enhanced their breast cancer screening practices [7]. Similar results have been described in a survey of attenders and non-attenders to mammography screening in Singapore and in an interview of 57 immigrants from India and Pakistan to Canada. They found that social support, especially from spouses could positively influence women’s breast cancer screening decisions [21,22]. Few men in our study mentioned that their wives are not used to leaving home without being escorted by the husband or older sons. Other studies have indicated that women’s cancer screening decisions can be inhibited by unsupportive spouses and suggested that breast health awareness interventions should include the targeting of the partners of women to enhance their supportive leverage towards breast cancer screening [23-26]. Gotay and Wilson [27] described four dimensions of social support that can enhance women’s screening behaviour: emotional, instrumental, appraisal and informational support. They suggested that family members can offer emotional support by being compassionate, showing concern, listening and establishing ties of trust. As for the instrumental support it can be in offering tangible assistance by giving time, money and effort. Informational support can take the form of counselling, guidance and advice. As for appraisal support, it affirms the woman’s status and feeling of self-value.

Men in this study expressed more support and compassion than the women’s perceptions as shown in our previous study [7]. On the other hand, they confirmed the women’s views that, if the wife gets breast cancer, the husband might have a negative attitude and some might reject the sick wife or start looking for a new one [7]. The interviewees perceived other men’s rejection of the wife if she gets breast cancer as a withdrawal strategy to avoid feeling helpless which could undermine the man’s role as the head of the family who is responsible for taking care of the sick wife. This is consistent with the findings of a literature review by Vlassoff [28] about gender and health which indicated that women with a chronic disease were less likely to receive support from their husbands. The marital obligations and needs as explained by the men in our study could to some extent be an expression of the socio-cultural constructs of gendered roles and obligations that they felt men in Jordan are bound to live up to. According to the literature men experience relatively greater social pressure than women to comply with gendered societal beliefs about masculinity including being independent, strong and in control [29-33].

The interviewees in our study talked about mastectomy as a consequence of breast cancer and few of them associated that with female body deformation and diminished femininity. Fear of the risk of death or becoming less of a woman and failing to meet the husband’s marital needs as a consequence of mastectomy was also voiced in the narratives of our previous study with Jordanian women [7]. Women’s fear from the consequences of screening and breast cancer diagnosis with regard to the potential risk of death or mastectomy, distorted body and partner’s abandonment was reported by Peek et al. [34] in his study about African-American women perceptions about breast cancer screening. Breast cancer affects the woman’s perceived body image and sexuality and that is strongly influenced by the perceptions of others [35,36]. Women may wrongly perceive that their partner will be repulsed by the women’s changed body image as a result of mastectomy [37].

The respondents voiced their concerns about breast cancer being a taboo subject that is not openly discussed in the society. Jordan’s tribal context highly values the collective good above the individual’s best interests. Thus, one might feel obliged to hide a stigmatizing illness to accommodate the expectations of others; to avoid embarrassment and to save the family’s reputation; especially if the disease is socially perceived to be inheritable [38]. The social stigma of cancer was also reported in our previous study with Jordanian women and in other studies [7,39,40]. Kawar (2012) reported embarrassment in talking about breast cancer in immigrant Jordanian and Palestinian women in USA [40]. Women viewed cancer as a shame label that could stigmatize the woman and her family. Studies have indicated that in the Mediterranean cultures, honour is centred on avoiding shameful behaviours and conserving the individual’s and the family’s good reputation from stigmatizing labels based on the masculine and feminine honour prescriptions of a socially interdependent culture [41].

The interviewees talked about the contribution of the awareness campaigns to their willingness to talk openly about breast cancer and to their readiness to face the culture of Eib (shame). Based on the Social Identity Theory (SIT) of Tajfel and Turner [42], individuals’ define themselves as members of a social group and strive to preserve a positive image within the group because the respect within the group enhances the individual member’s self-value. Social stigma as explained by Erving Goffman [43] is an attribute or a behaviour which leads to a reaction by other members within the social group that affects the normal identity of the stigmatized member. A critical review by Pasick & Burke [44] highlighted the importance of the social context as a dynamic shaper of individual’s beliefs and experiences and recommended using multilevel interventions that integrate social and individual approaches in promoting breast cancer screening. Based on a literature review of published articles, Randolph & Viswanath [45] concluded that effective health promotion campaigns should ensure sufficient exposure of the audience to carefully tailored appropriate messages and be able to create a supportive social environment that allows the target audience to make the recommended behavioural change. In addition, they suggested that the campaigns must be based on careful understanding of the determinants of health behaviour that could potentially lead to the desired health outcome.

The results of this study could be transferable to other similar contexts; however, they should be interpreted within the context of several limitations and cannot be generalized to all Jordanian or Arabic men. Although our pilot interviews showed that men felt more comfortable talking to the female interviewer about the breast health issues of the females within their family, still, there is a potential that the findings of this study might have been affected by social desirability bias. One could argue that the participants might have provided answers that they believed the female interviewer wanted to hear. To avoid this potential problem, the interviewer attempted to remain open-minded and unbiased and avoided leading questions.

Our findings were focused on the husband’s role in the wife’s breast health. We recruited 24 married men and 4 single men, because we assumed that married men could give us a wider span of experiences related to their contribution to the breast health of the females within their families. However, the majority of the interviewees responded primarily about the wife and when the interviewer probed about their role in the breast health of other female family members, the narratives exposed the culture of Eib.

There is an apparent contradiction in the data with regard to the respondents’ own supportive perceptions and attitudes versus other men’s rejection of a wife inflicted by breast cancer. However, this cannot be interpreted as a sign of on-going ambivalence or a shift in social norms because the interviewees were purposely selected based on not having any females within their direct family with a previous diagnosis of breast cancer. Thus, their contextual views about husband’s rejection of breast cancer inflicted wife don’t reflect their own reaction if they themselves experienced having a wife with breast cancer. This necessitates further qualitative research that explores the perceptions of husbands’ of breast cancer inflicted wives in Jordan.

As far as we know this is the first study to explore Jordanian men’s individual and contextual perspectives on women’s breast cancer and their role in the breast health of the females within their families. We did not provide any monetary compensation for the interviewees in this study. There is a possibility that those who voluntary decided to participate were interested in expressing their views about breast cancer since it is a topic that is not openly discussed in public. The interviewees were engaged and after the interview they sought to get answers to their inquiries.

The strengths of this study are in its design including the purposive recruitment of interviewees with different ages from four governorates in Jordan which contributed to the richness of data, the triangulation of the researchers who thoroughly reviewed the transcripts and the codes and contributed to the thematic interpretation [46]. The researchers brought rigour into the analytical process because of their different qualifications, experiences and familiarity with the Jordanian context. Previous engagement of the authors in public health research in Jordan and the Middle East might have also enhanced the trustworthiness of this research [47].

## Conclusions

Our findings confirm that the perceptions and attitudes of men towards breast cancer and breast health are fundamental and integral for the development of effective breast health promotion strategies. We conclude that Jordanian men perceive themselves as having a positive role in supporting, guiding and encouraging their wives to follow breast cancer early detection recommendations. Thus, breast health awareness interventions should be gender conscious and should involve husbands to capitalize on family support.

## Competing interests

The authors declare that they have no competing interests.

## Authors’ contributions

HT, RQ and RW conceived the study; HT performed data collection, analysis, and drafted the manuscript. VB, RQ, RW and LN participated in the data analysis and contributed intellectually to the writing of the manuscript. All the authors read and approved the final manuscript.

## Pre-publication history

The pre-publication history for this paper can be accessed here:

http://www.biomedcentral.com/1472-6874/13/41/prepub
